# Apnée lors d'une intubation difficile prévisible pour un volumineux kyste laryngé

**DOI:** 10.11604/pamj.2014.19.123.3748

**Published:** 2014-10-03

**Authors:** Mohammed Zalagh, Moulay Ahmed Hachimi, Ali Boukhari, Hicham Attifi, Mounir Hmidi, Abdelhamid Messary

**Affiliations:** 1 Service d'Otorhinolaryngologie, et de Chirurgie Cervico-Faciale, Hôpital Militaire Moulay Ismaïl, Meknès, Maroc; 2 Service de Réanimation et d'Anesthésie, Hôpital Militaire Moulay Ismaïl, Meknès, Maroc

**Keywords:** Apnée, intubation difficile, kyste laryngé, Apnea, difficult intubation, laryngeal cyst

## Abstract

Les auteurs présentent un cas d'obstruction aiguë des voies aériennes supérieures au moment de la tentative d'une intubation endotrachéale difficile prévisible en rapport avec un volumineux kyste du larynx. A travers ce cas clinique, les auteurs insistent sur la coopération étroite entre médecin anesthésiste et chirurgien ORL en termes d’échanges d'informations pré-opératoires, en particulier les données de la fibroscopie et la tomodensitométrie.

## Introduction

Les kystes laryngés sont rares; mais peuvent entraîner une obstruction sévère des voies aériennes supérieures, voire le décès. Arens et al. Ont classé les kystes laryngés selon leur localisation: les cordes vocales (58,2%), repli ventriculaire (18,3%), la vallécule (10,5%), l’épiglotte (10,1%) et le repli aryépiglottique (2,2). Ils ont distingué le kyste laryngé congénital, rétentionnel et à inclusion [1]. Les signes cliniques typiques sont représentés par le stridor, la dysphagie et la détresse respiratoire à des degrés variables. Leur traitement est chirurgical, excision complète ou marsupialisation. Les auteurs présentent un cas d'obstruction aiguë des voies aériennes supérieures au moment de la tentative d'une intubation endotrachéale difficile prévisible en rapport avec un volumineux kyste du larynx.

## Patient et observation

Une jeune fille de 22 ans, coopérante, a été admise dans notre service pour cure chirurgicale d'un kyste laryngé obstructif. Son histoire clinique a été marquée par la survenue d'une sensation de corps étranger dans la gorge avec une dysphagie modérée, une dyspnée et des épisodes d'apnée obstructive au moment de l'inspiration et le décubitus dorsal. La position assise ou debout, le décubitus ventral faisaient disparaître la dyspnée et l'apnée.

**Figure 1:**
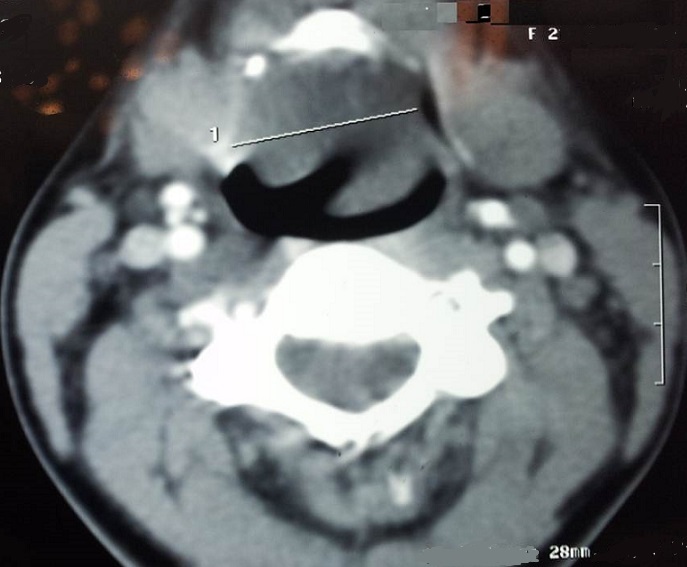
TDM en coupe axiale montrant le kyste laryngé

**Figure 2:**
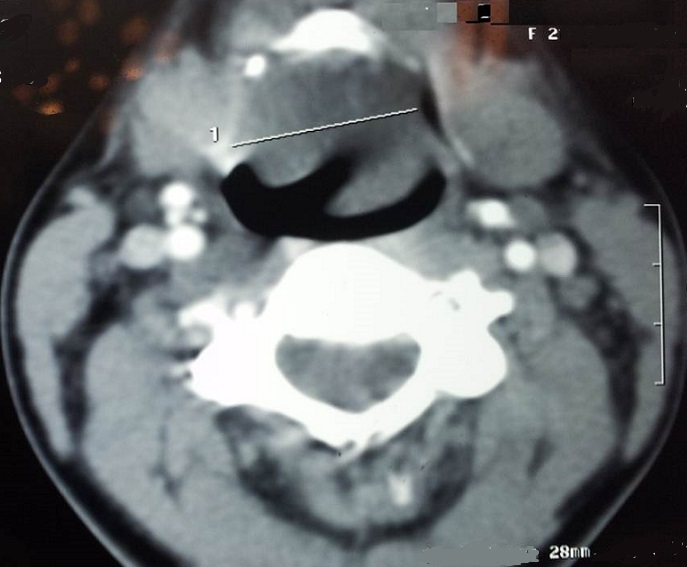
Apnée lors d'une intubation difficile prévisible pour un volumineux kyste laryngé

La fibroscopie pharyngo-laryngée en position assise a mis en évidence la présence d'une volumineuse formation arrondie réduisant massivement la lumière pharyngo-laryngée entravant la visibilité de la glotte. Sa surface est régulière et lisse; tandis que sa consistance est molle à l'attouchement par le bout du fibroscope. La TDM cervicale (Figure 1, Figure 2) a objectivé une masse de nature kystique, émergeant du bord latéral de l’épiglotte et du repli ary-épiglotique gauches, mesurant environ 3 cm de grand diamètre. L'examen à visée pré-anesthésique a montré une bonne ouverture buccale avec un Mallanpati classe 1.

S'agissant d'une intubation difficile prévisible, nous avons décidé de réaliser une intubation endotrachéale de la patiente au fibroscope souple au moment de la ventilation spontanée. La patiente informée des tenants et des aboutissants de cette technique, notamment d'une éventuelle trachéotomie expéditive, a donné son consentement éclairé. Une prémédication a été prescrite à base d'Hydroxyzine la veille de la procédure et le matin au réveil. A son arrivée à la salle d'opération, un monitorage non invasif a été mis en place et la position adéquate pour une intubation éveillée au fibroscope a été adoptée. Nous avons fait pencher en avant et à gauche la tête de la patiente pour optimiser l'exposition endolaryngée et ne pas aggraver le stridor.

L'anesthésie des cavités nasales a été assurée par l'application de mèches imbibées de Lidocaïne^®^ 5% à la Naphazoline laissées au contact de la muqueuse nasale pendant 10 min. Celle de l'oropharynx par nébulisation à la Lidoocaïne^®^ 2% spray, suivie d'un gargarisme. Quant à l'anesthésie du pharyngo-larynx et de la trachée a été réalisée par instillations de Lidocaïne 2% à travers le canal aspirateur. Au niveau du pharyngo-larynx, la visualisation endoscopique de la glotte était impossible car le kyste ne laissait qu'un pertuis postéro-latéral droit. Nous nous sommes aidés vainement des man'uvres laryngées externes et de l'inclinaison de la tête de la patiente du côté gauche et en avant pour faire progresser le bronchoscope. Chemin faisant, le brochoscope a buté sur le kyste l'entraînant dans le vestibule laryngé où il s'est enclavé occasionnant une apnée résistante aux essais d'extraction par les efforts répétés d'expulsion du kyste par la patiente et l'utilisation de pince de Magill. En suspendant la patiente par le laryngoscope (Macintosh), l'ouverture expéditive du kyste et l'aspiration de son contenu liquide a permis son affaissement. La voie aérienne a été finalement libérée et l'intubation était alors possible en faisant passer une sonde d'intubation endotrachéale armée à ballonnet de 6 mm de diamètre. La SpO2 a remonté à 100%.

L'excision du tissu résiduel du kyste à partir de sa base d'implantation a été réalisée sous laryngoscopie directe en suspension par des micro ciseaux laryngés. L'amélioration clinique a été rapide avec disparition des symptômes décrits précédemment en post- opératoire immédiat. La patiente a été mise sous antibioprophylaxie, corticoïde et antalgique. L'examen histopathologique a confirmé la présence d'un kyste rétentionnel, sans signes de malignité.

## Discussion

Les Kystes laryngés, par leur localisation ou volume, peuvent poser un problème d'intubation classique. L'intubation fibroscopique est recommandée dans l'algorithme de l'intubation difficile [2, 3]. Cette approche des voies aériennes nécessite une formation supervisée, un niveau d'anesthésie adapté respectant la respiration spontanée et la maitrise de la technique fibroscopique [3, 4]. L'intubation vigile au fibroscope est réussie dans 88-100% des patients présentant une intubation difficile [2]. Cette option peut être restreinte chez les patients non coopérants et les enfants [2]. Les sédatifs et les hypnotiques sont bannis dans les intubations fibroscopiques vigiles avant de s'assurer que la sonde d'intubation est dans la trachée [4]. Une information préalable sur la position de la tête adéquate à la respiration permettrait de déterminer la position idéale qui pourrait minimiser le degré de l'obstruction dynamique des voies aériennes au cours de l'anesthésie, en particulier en cas de présence d'obstacle mécanique à ce niveau et faciliterait une intubation difficile.

Les patients avec une histoire de ronchopathie, apnée obstructive, obésité, hypoplasie mandibulaire ou obstruction nasale, ou ceux avec une hyperplasie des amygdales palatines sont spécialement vulnérables au collapsus des voies aériennes durant l'anesthésie [5]. La ventilation au masque peut donc être impossible et il faut se préparer à une trachéotomie chez ces patients. La chute du kyste dans le vestibule laryngé chez notre patiente peut être justifiée par le coup infligé par le bout du bronchoscope au kyste favorisé par l'effet de pesanteur sur la masse du kystique. L'enclavement du kyste dans le vestibule et sa résistance aux man'uvres de désenclavement peuvent être expliquées par la configuration anatomique du vestibule épousant le kyste malléable et tendu et le rôle sphinctérien de la margelle laryngée. Chez notre patient, l'ouverture du kyste et l'aspiration de son contenu liquide a permis d'achever l'intubation endotrachéale d’éviter une trachéotomie de sauvetage. Les auteurs recommandent cette attitude devant toute formation kystique volumineuse potentiellement obstructive.

## Conclusion

Le médecin anesthésiste et le chirurgien ORL doivent coopérer en parfaite harmonie et conjuguer leur informations. Ils doivent disposer d'une stratégie pré formulée devant une intubation difficile [2] en présence d'une pathologie obstructive des vois aériennes supérieures. Ils doivent identifier les approches alternatives qui peuvent être utilisées en cas d’échec de la première approche ou quand celle-ci n'est pas faisable [2]. Les données de la fibroscopie et la bonne lecture de la TDM préopératoires sont indispensables à la détermination de la nature, de la localisation et du volume d'une formation tissulaire occupant les voies aériennes.
